# Opinion amplification causes extreme polarization in social networks

**DOI:** 10.1038/s41598-022-22856-z

**Published:** 2022-10-28

**Authors:** Soo Ling Lim, Peter J. Bentley

**Affiliations:** 1 Department of Computer Science, University College London, London, UK; 2 Autodesk Research, London, UK

**Keywords:** Computational science, Computer science, Human behaviour

## Abstract

Extreme polarization of opinions fuels many of the problems facing our societies today, from issues on human rights to the environment. Social media provides the vehicle for these opinions and enables the spread of ideas faster than ever before. Previous computational models have suggested that significant external events can induce extreme polarization. We introduce the Social Opinion Amplification Model (SOAM) to investigate an alternative hypothesis: that *opinion amplification* can result in extreme polarization. SOAM models effects such as sensationalism, hype, or “fake news” as people express amplified versions of their actual opinions, motivated by the desire to gain a greater following. We show for the first time that this simple idea results in extreme polarization, especially when the degree of amplification is small. We further show that such extreme polarization can be prevented by two methods: preventing individuals from amplifying more than five times, or through consistent dissemination of balanced opinions to the population. It is natural to try and have the loudest voice in a crowd when we seek attention; this work suggests that instead of shouting to be heard and generating an uproar, it is better for all if we speak with moderation.

## Introduction

Polarization of opinions on social networks is increasingly evident today, with highly contrasting beliefs being shared in politics, environmental and social issues. The likely repercussions of polarization in our societies are well established, from damaging the democratic process to a decrease in tolerance for others^1^. With so much at stake, the use of computational models to understand the causes of polarization is receiving more attention.

When using models to study social influences, it is common for opinions to converge towards a consensus, or fragment into two or more clusters^2^. In both cases, final opinions fall within the initial range of opinions. Yet social media breeds not just polarized opinions, but *extreme* opinions, that might otherwise be considered outliers in population norms. Thus in our models, a key outcome to study is when individuals influence each other such that opinions diverge towards extremes that are outside their initial range of opinions.

Synchronized external events have been shown to be a possible cause of polarization^2^. However, it is possible that polarization happens gradually in the network without needing external intervention. In social media, it is common for people to amplify what they actually feel about something, in order to attract attention, because the more extreme a post, the more popular it is^1,3^. We use the term *opinion amplification* to encompass the range of behaviors by users that may distort the original opinion with a more positive or negative sentiment. Such behaviors include making unfounded assumptions, making generalizations or summaries, selectively quoting, editorializing, or misunderstanding^3^.

Opinion amplification may happen at a low level at all times, but it proliferates across a network once a topic is trending^4^. Here we look at opinion amplification as potential cause for polarization, focusing specifically on extreme polarization. This differs fundamentally from previous work as previous models investigated polarization that results from interactions between individuals, and more recently events that are external to the individuals themselves; in this work we argue that noise fails to model this crucial phenomenon. We hypothesize that opinions can become biased towards a more positive or negative sentiment through opinion amplification. We propose the Social Opinion Amplification Model (SOAM) to investigate these ideas. We remove well-studied variables such as noise from the model in order to identify the minimum features needed to create extreme polarization in a population through opinion amplification.

## Background

The inexorable draw of expressing extreme sentiments may first have emerged in conventional media. It is well known that health research claims become exaggerated in press releases and news articles, with more than 50% of the press releases from certain universities exaggerated, and some news agencies exaggerating about 60% of the articles they publish^5^. This is “spin”, defined as specific reporting strategies, intentional or unintentional, that can emphasize the beneficial effect of the experimental treatment^6^. “Sensationalism” is a close bedfellow when reporting general topics—a discourse strategy of “packaging” information in news headlines in such a way that news items are presented as more interesting, extraordinary and relevant^7^. These practises became ever more prevalent as the competition for online customers increased, becoming refined into new genres such as “clickbaits”—nothing but amplified headlines designed to lure the readers to click on the link^8^, and hype in online reviews, where the hyped review is always absolute positive or negative^9^.

As conventional media has transitioned to social media, today everyone is a “media outlet”, so the lure of attention-seeking behavior is now felt by individuals. Influencers have used beauty filters to make the products they are advertising appear more effective, resulting in warnings by Advertising Standards Agency (ASA)^10^. Young people aged 11–18 were observed to exaggerate their behaviors as they aimed to live up to amplified claims about popularity^11^. Even the accidental use of certain words can make readers believe causal relationships that may not exist^12^. This is also known as sentiment polarity, an important feature in fake news—in order to make their news persuasive, authors often express strong positive or negative feeling in the content^13,14^. The result is that bizarre conspiracy theories that might once have been the domain of a tiny minority are now routinely given the same credence as evidence-backed science by large portions of the population^15^.

It is infeasible to perform experiments on real human populations in order to understand causation of extreme polarization. Computational models provide an essential tool to overcome this empirical limitation. Computational models have been used for decades to study opinion dynamics, with early works often focused on consensus formation^16–18^. Deffuant et al. developed a model of opinion dynamics where convergence of opinions into one average opinion and convergence into multiple opinion clusters are observed^19^. Their model consists of a population of $N$ agents $i$ with continuous opinions ${x}_{i}$. At each timestep, two randomly chosen agents “meet” and they re-adjust their opinion when their difference of opinion is smaller in magnitude than a threshold $\varepsilon$. Suppose that the two agents have opinion $x$ and $x^{\prime}$ and that $\left| {x - x^{\prime} } \right| < \varepsilon$, opinions are then adjusted according to:

1 $$x = x + \mu \cdot \left( {x^{\prime} - x} \right)\;{\text{and}}\;x^{\prime} = x^{\prime} + \mu \cdot \left( {x^{\prime} - x} \right)$$

where $\mu$ is the convergence parameter taken between 0 and 0.5 during the simulations. Deffuant et al. found that the value of $\varepsilon$ is the main influencer on the dynamics of the model, when it is high, convergence into one opinion occurs, and when it is low, polarization/fragmentation occurs (convergence into multiple opinions)^19^. $\mu$ and $N$ only influence convergence time and the distribution of final opinions. They applied their model to a social network of agents, whereby any agent in the model can only interact with 4 connected neighbors on a grid (so that the random selection of agents to interact can only come from connected neighbors) and found the same results.

Hegselmann and Krause developed a model with bounded confidence to investigate opinion fragmentation in which consensus and polarization are special cases^20^. The Hegselmann-Krause (HK) model is defined as:

2 $$x_{i} \left( {t + 1} \right) = \left| { I\left( {i,x\left( t \right)} \right)} \right|^{ - 1} \mathop \sum \limits_{{j \in I\left( {i,x\left( t \right)} \right)}} x_{j} \left( t \right)\;{\text{for}}\; t \in T$$

where $I\left( {i,x} \right) = \{ 1 \le j \le n| \left|x_{i} - x_{j} \right| \le \varepsilon_{i} \}$ and $\varepsilon_{i} \ge 0$ is the confidence level of agent $i$. Agent $i$ takes only those agents $j$ into account whose opinions differ from his own by not more than ${\varepsilon }_{i}$. The base case assumes a uniform level of confidence, i.e., ${\varepsilon }_{i}=\varepsilon$ for all agents $i$. The authors found that higher values of confidence threshold $\varepsilon$ lead to consensus, while lower values lead to polarization and fragmentation. In all their runs, regardless of whether consensus or polarization occurs, the range of opinions decreases as the simulation runs.

Fu et al. modified the HK model by dividing the population into open-minded, moderate-minded and closed-minded agents^21^. They found that the number of final opinion clusters is dominated by the closed-minded agents; open-minded agents cannot contribute to forming opinion consensus and the existence of open-minded agents may diversify the final opinions.

Cheng and Yu suggested that in many social situations, an individual’s opinion formation and expression may be different because the individual feels pressured to express an opinion similar to the public opinion in the group^22^. They propose a bounded confidence plus group pressure model, in which each individual forms an inner opinion relative to the bound of confidence and expresses an opinion, taking group pressure into consideration. A group with all individuals facing group pressure always reach a consensus. In a mixed group with both pressured and non-pressured individuals, the consensus threshold ε is significantly reduced, and group pressure does not always help to promote consensus; although similar to other models, in their work polarization does not occur.

**Figure 1:**
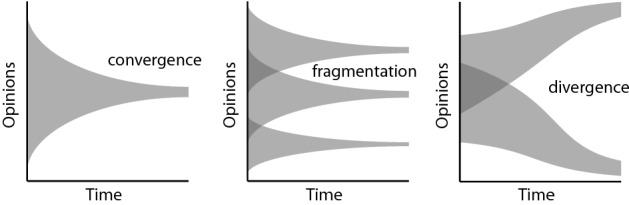
Assimilative influence (left), bounded assimilative influence (middle) and differentiative influence (right)^2^.

Most recently, Condie and Condie classified social influence into assimilative and differentiative^2^. Assimilative influence occurs when opinions converge towards a consensus, or fragment into two or more converging clusters, all within the initial range of opinions. Differentiative influence—the focus of our work—occurs when individuals with very dissimilar opinions can influence each other causing divergence towards extreme opinions (see Fig. 1). Condie and Condie proposed the Social Influence and Event Model (SIEM)^2^ which builds on the HK bounded confidence model, with $\varepsilon$ as confidence threshold, with the following main differences: (1) agents form a social network, (2) an individual $i$ will only change their opinion if their certainty, ${C}_{j,t}\in [\mathrm{0,1}]$, is less than the average certainty of other individuals with which they interact at time $t$, (3) most importantly, events can influence many individuals synchronistically over a limited period of time. Events can have a large impact on the distribution of opinions because their influence acts synchronistically across a large proportion of the population, whereas an individual can only interact with small numbers of other individuals at any particular time. The simulation results showed that SEIM without events exhibited the range of behaviors generated by other influence models under differing levels of confidence threshold $\varepsilon$ leading to consensus (or assimilative influence in their definition). With the presence of strong events, when the confidence threshold $\varepsilon$ is high (low homophily), opinions swing between extremes, and when the confidence threshold $\varepsilon$ is low (high homophily), opinions diverged into extremes. Condie and Condie^2^ also introduced a measure of *conflict*, $\Delta {O}_{t}=SD({O}_{i,t})$, in the population which they defined as the standard deviation of individual opinions ${O}_{i,t}$ across the population at timestep $t$.

Building further on these ideas, Macy et al. used a general model of opinion dynamics to demonstrate the existence of tipping points, at which even an external threat, such as a global pandemic, economic collapse, may be insufficient to reverse the self-reinforcing dynamics of partisan polarization^23^. Agents in the model have initially random locations in a multidimensional issue space consisting of membership in one of two equal-sized parties and positions on 10 issues. Agents then update their issue positions by moving closer to nearby neighbors and farther from those with whom they disagree, depending on the agents’ tolerance of disagreement and strength of party identification compared to their ideological commitment to the issues. They manipulated agents’ tolerance for disagreement and strength of party identification, and introduced exogenous shocks that corresponds to events (following Condie and Condie^2^) that create a shared interest against a common threat (e.g., a global pandemic).

These works all demonstrate the value of this form of modelling to explore opinion dynamics, while assuming expressed opinion is same as actual opinion.

## Methods

As explored in the previous section, people may express more extreme opinions on social media compared to their own internal beliefs, and we hypothesize that this may cause influence across the population towards a more positive or negative sentiment. We incorporate this notion of an agent presenting an amplified version of their own opinion in our model, which is built on the Hegselmann–Krause (HK) bounded confidence model of opinion formation.

The Social Opinion Amplification Model (SOAM) consists of a network of individuals, where individuals can influence other individuals that they are connected to on the social network in relation to a specific issue. Opinions are continuous and individuals influence each other in each timestep.

The key innovation in our model is the concept of an *expressed opinion*, which for individuals who have a tendency to amplify, is stronger than the individual’s actual opinion. This is backed up by early theories that online opinion expression does not necessarily reflect an individual’s actual opinion^24^ and recent literature that people actually express stronger opinions on social media, compared to actual truth^3^ or hold different public and private opinions^25^.

We make a random directed network, the most common network structure used to build synthetic social networks^26^. The use of directed networks enables the representation of asymmetric relationships—individual A may affect individual B but the reverse may not be true (in online social networks this might correspond to B following A without reciprocation, resulting in A influencing B, but B not influencing A). The network comprises $k$ average links per node; the entire network is considered, including any subnetworks following Condie and Condie^2^.

An individual $i$ in the network at timestep $t=0$ has an initial opinion ${O}_{i,t=0}$.

The opinion of an individual $i$ at timestep $t>0$ is defined as:

3 $$O_{i,t} = \left( {O_{i,t - 1} + \mathop \sum \limits_{{j \in {\varvec{I}}_{i,t} }} SO_{j,t} } \right)/\left( {1 + \left| {{\varvec{I}}_{i,t} } \right|} \right)$$

where ${O}_{i,t-1}$ is individual $i$’s opinion in the previous timestep, ${{\varvec{I}}}_{i,t}$ is the set of individuals connected to individual $i$, whose *expressed opinion* is within the confidence threshold $\varepsilon$, as per Hegselmann and Krause^20^:

4 $${\varvec{I}}_{i,t} = \{ j| \left| {O_{i,t - 1} - SO_{j,t - 1} } \right| \le \varepsilon \}$$

An individual $i$’s *expressed opinion* is calculated as follows:

5 $$SO_{i,t} = \left\{ {\begin{array}{*{20}l} {\left\{ {\begin{array}{*{20}c} {O_{i,t - 1} - \sigma_{i,t} , if \,\,O_{i,t - 1} < 0} \\ {O_{i,t - 1} + \sigma_{i,t} , if\,\, O_{i,t - 1} \ge 0} \\ \end{array} } \right.} \hfill & {if\;E_{i,t} = True} \hfill \\ {O_{i,t - 1} } \hfill & {otherwise} \hfill \\ \end{array} } \right.$$

where ${E}_{i,t}$ is whether individual $i$ will amplify its opinion at timestep $t$, and ${\sigma }_{i,t}$ is the individual’s amplified amount at timestep $t$.

Table 1 provides the definitions for SOAM variables at the individual level and Table 2 provides the definitions for SOAM variables at the system level. Finally, Table 3 compares the main features of SOAM with the features of similar models in the literature to illustrate the similarities and differences and justify our design decisions.

**Table 1:** SOAM individual level variables and values.

Variable	Definition	Value range
${O}_{i,t=0}$	Initialized opinion of individual $i$ at timestep 0, which is a random number between value range. A value of −1.0 means a very negative opinion on the topic, and a value of 1 means a very positive opinion on the topic. Note that for $t$ > 0, there is potential for opinions to go beyond the original opinion range	[−1.0, 1.0]
${O}_{i,t>0}$	Opinion of individual $i$ at timestep $t$ > 0	[−∞, ∞]
${E}_{i}$	Whether or not the individual $i$ is an amplifier	True/False
${E}_{i,t}$	Whether or not the individual $i$ amplifies their opinion in timestep $t$, which is True if ${E}_{i}=True\, and\, {P}_{i,t}\le Pe,$ where ${P}_{i,t}$ is a generated probability for that individual $i$ at that timestep $t$	True/False
${\sigma }_{i,t}$	Amplified amount for individual $i$ at timestep $t$, a random number between 0 and $s$, where $s$ is the strength of amplification, see Table 2	[0, $s$]

**Table 2:** SOAM system level variables and values.

Variable	Definition	Value range
$t$	Timesteps	[0, ∞]
$n$	Number of nodes	[0, ∞]
$k$	Average links per node	[0, n]
$\varepsilon$	Confidence threshold	[0, 1]
$\pi$	Proportion of the population who are amplifiers	[0, 1]
$p$	Probability of amplifiers amplifying opinions	[0, 1]
$s$	Strength of amplification	[0, 1]

**Table 3:** SOAM features compared to literature.

Model	Social network	How opinion is expressed	Opinion update	Influence
HK bounded confidence model^20^	Fully connected	As is. What the individual thinks and what the individual expresses are the same	Influence occurs only if opinions are not too far from each other (within confidence threshold $\varepsilon$)	Each individual influences all the other individuals
Social Influence and Event Model^2^	Random network with average link per individual of $k$ = 5. A new network is formed every timestep	As is. What the individual thinks and what the individual expresses are the same	An individual will only change their opinion if their certainty is less than the average certainty of other individuals with which they interact at timestep $t$ An individual is influenced by an event if the event strength is not too far from the individual’s opinion (within $\varepsilon$) and the individual’s confidence is less than or equal to the event strength	Influence occurs between linked individuals
SOAM	Random network with $k$ = 2, 5 or 10 average links per individual	Some individuals provide *expressed opinions* that are amplified versions of their actual opinions	Influence occurs only if opinions are not too far from each other (within $\varepsilon$)	Influence occurs between linked individuals

We only compare with models that SOAM is based on.

## Results

Given the hypothesis that amplified individual opinions can cause polarization, we study the effect of amplification on opinion dynamics under different confidence thresholds. We compare the model baseline without amplification with the model using amplification. In more detail:

1. No amplification, for confidence thresholds $\varepsilon$ = 0.2 and 0.8. These confidence threshold settings follow the range of settings that was explored in Condie and Condie^2^. Confidence threshold determines the range in which an individual will re-adjust their opinion, i.e., an individual will re-adjust their opinion based on other individual’s opinions if the difference of opinion is smaller in magnitude than the confidence threshold, so a low confidence threshold means that the individuals are less likely to re-adjust their opinions.
2. Amplification, where the proportion of the population who are amplifiers $\pi$ = 0.2 (low proportion of amplifiers) and 0.5 (high proportion of amplifiers), amplification probability $p$ = 0.5, amplification strength $s$ = 0.5, for confidence thresholds $\varepsilon$ = 0.2 and 0.8.

**Figure 2:**
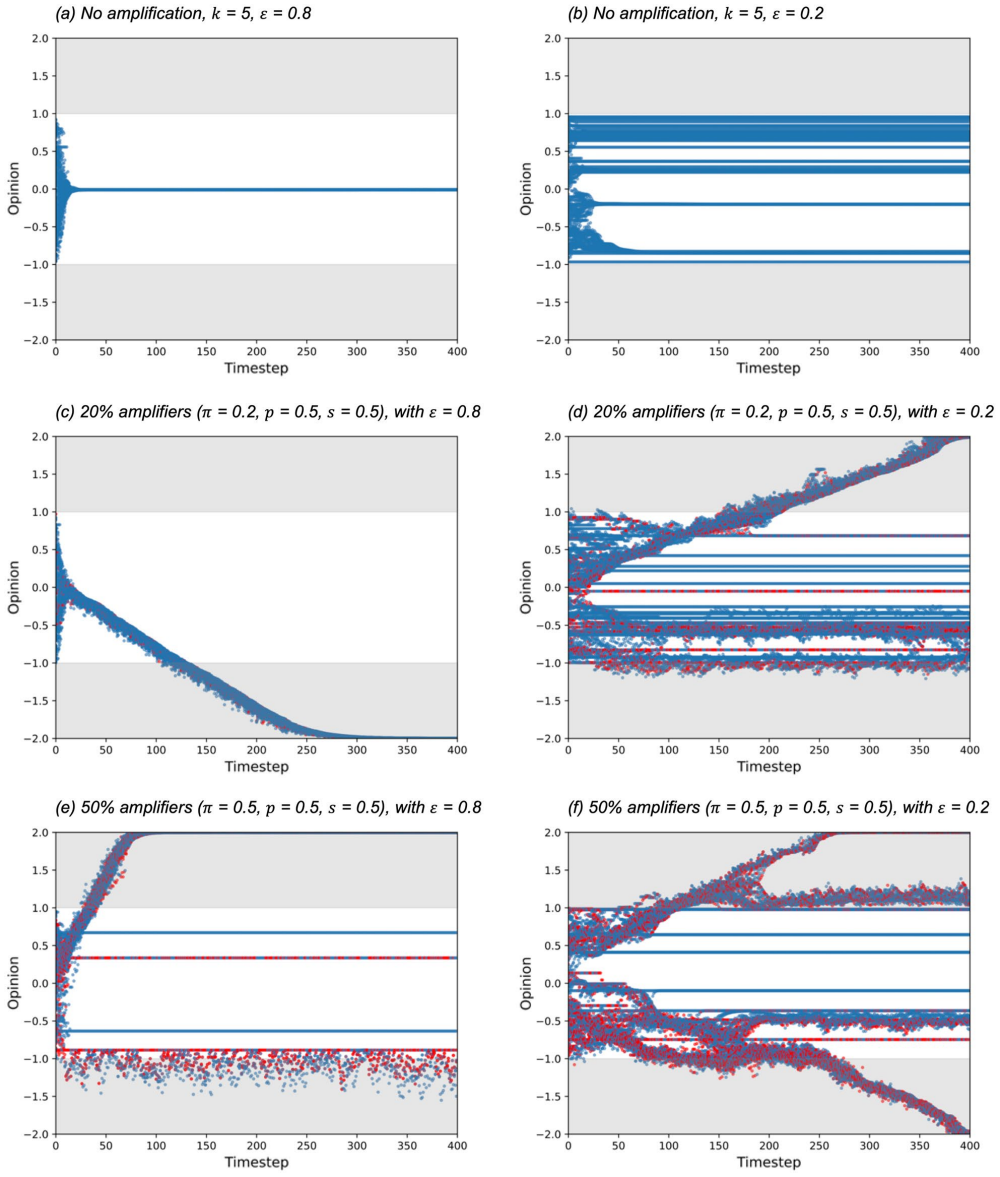
Opinions of individuals starting from a random distribution [−1.0, 1.0] under a range of conditions. Red dots denote individuals who are amplifying in that timestep. Grey areas indicate opinions outside the initial opinion range. Y-axis shown from −2.0 to 2.0 (double the initial opinion range) for clarity; in (**c**) to (**f**), opinions exceed this range and become even more extreme.

We ran the model for number of timesteps $t$ = 400, number of nodes $n$ = 100, and average links per node $k$ = 5, and plotted each individual’s opinion over time. Results were invariant to the number of nodes for tests up to $n$ = 1000. For clarity our plot scale ranges from −2.0 to 2.0 (doubling the initial opinion range), in order to show extreme polarization if it exists. We plot the actual opinions and not the expressed opinions in this work as they indicate the degree to which population opinions are truly modified—while we may all exaggerate at times, our actions are determined by our true beliefs. Our results show that when there are no amplifiers, we see the usual convergence and fragmented convergence, and the opinion range is always within the initial range. In other words, assimilative influence (Fig. 1(left)) and bounded assimilative influence (Fig. 1(middle)) occurred in Fig. 2a,b respectively. When there is amplification ($\pi$ = 0.2 and 0.5), we observe that extreme polarization illustrated in Fig. 1(right) occurs, see Fig. 2c–f. When $\varepsilon$ = 0.8, extreme polarization tends to occur in a single direction (e.g., Fig. 2c shows convergence to extreme negative sentiment and Fig. 2e shows convergence to extreme positive sentiment), while $\varepsilon$ = 0.2 results in multiple convergences of clusters, some extreme some not, see Fig. 2e,f. When $\varepsilon$ = 0.2 and proportion of amplifiers is 50% ($\pi$ = 0.5), extreme polarization occurs in both positive and negative sentiments Fig. 2f. Note that the polarization more than doubles the initial opinion sentiment values, showing extreme sentiments beyond 2.0 or lower than −2.0 (our Fig. 2 opinion plot scale is from −2.0 to 2.0).

SOAM illustrates that when individuals amplify their opinion, extreme polarization occurs, and when the proportion of amplifiers are higher, extreme polarization occurs quicker and at a bigger magnitude. Although our work here focuses on directed networks, the same findings are evident for undirected networks. To understand in more detail how variations in the parameters relating to amplification can affect the development of the distribution of population opinion across time, we ran SOAM under a range of different variables and settings:

- *Baseline: Confidence threshold no amplification*. How does different confidence thresholds, $\varepsilon$, affect conflict when there is no amplification? $k$ = 5, for confidence thresholds $\varepsilon$ = 0.2, 0.5 and 0.8.
- *Links per node no amplification*. How does the different average number of links per node, $k$, affect conflict when there is no amplification? $k$ = 2, 5 and 10, for confidence threshold $\varepsilon$=0.8, comparing with Baseline.
- *Confidence threshold with amplification*. How do different confidence thresholds, $\varepsilon$, affect conflict when there is a low proportion of amplifiers? $\pi$ = 0.2, $p$ = 0.5, $s$ = 0.2, $k$ = 5, for confidence thresholds $\varepsilon$ = 0.2, 0.5 and 0.8, comparing with Baseline.
- *Strength of amplification*. How does strength of amplification, $s$, affect the results? $k$ = 5, $\varepsilon$ = 0.2, $\pi$ = 0.2, $p$ = 0.5, $s$ = 0.2, 0.5 and 0.8, comparing with Baseline.
- *Proportion of amplifiers*. How does proportion of amplifiers, $\pi$, affect the results? $k$ = 5, $\varepsilon$ = 0.2, $p$ = 0.5, $s$ = 0.2, $\pi$ = 0.2, 0.5 and 0.8, comparing with Baseline.
- *Probability of amplification*. How does probability of amplifiers amplifying opinions, $p$, affect the results? $k$ = 5, $\varepsilon$ = 0.2, $\pi$ = 0.2, $s$ = 0.2, $p$ = 0.2, 0.5 and 0.8, comparing with Baseline.

Similar to the previous experiment, we ran the model for number of timesteps $t$ = 400, and for the number of nodes $n$ = 100. We measure and plot population conflict over time, defined by Condie and Condie^2^ as the standard deviation of population opinion $\Delta {O}_{t}=SD({O}_{i,t})$, where ${O}_{i,t}$ is the individual’s opinion at timestep $t$. Conflict is a useful measure when we wish to understand the diversity of opinions in the population, with higher conflict indicative of a broader range (which may be the result of polarization).

**Figure 3:**
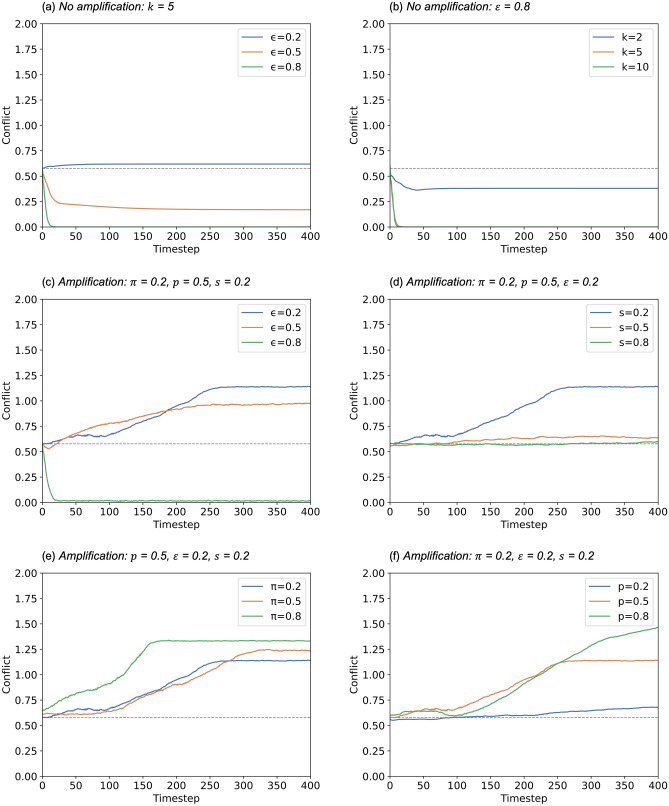
Conflict ($\Delta {O}_{t}$) tracked over 400 timesteps, starting from a uniform random distribution of opinions ($\Delta {O}_{t}=1/\sqrt{3}=0.577$), indicated with a grey dotted line in each chart. Shown are dependencies on: (**a**) confidence threshold when there were no amplifications; (**b**) average number of connections per individual per timestep when there were no amplifications; (**c**) confidence threshold when there were amplifications; (**d**) strength of amplification; (**e**) proportion of amplifiers; and (**f**) probability of amplification.

Our results show that conflict levels decline rapidly when confidence threshold $\varepsilon$ is high, see Fig. 3a when $\varepsilon$ = 0.8, conflict reached a level of zero very early on in the run, which is also consistent with the results in Fig. 2a. And vice versa, low confidence threshold $\varepsilon$ = 0.2 results in highest conflict among the three thresholds, although without amplification, the level of conflict is only slightly above the random opinion distribution at $t$ = 0. Reducing the average number of links per node increases conflict, Fig. 3b shows that with a very low average number of links per node of $k$ = 2, conflict remains steady at around 0.3, while $k$ = 5 and 10, conflict reduces to zero. We can see that in Fig. 3c, lower confidence threshold results in higher conflict, this similar to Fig. 3a where there is no amplification. When there is amplification, low and medium levels of confidence threshold $\varepsilon$ = 0.2 and 0.5, results in conflict levels that continue to increase as time progresses, reaching approximately 1.0 for $\varepsilon$ = 0.5 and 1.25 for $\varepsilon$ = 0.2. In Fig. 3d, $s$ = 0.2 is the only setting where conflict increases over time, while $s$ = 0.5 and 0.8 maintains conflict as the same level as the start, this is because a high strength of amplification may make the opinion so extreme that others can no longer relate to it (it falls outside the confidence threshold) and so no longer influences others. Increasing the proportion of amplifiers increases conflict, with $\pi$ = 0.8 more than double the original conflict level at $t$ = 150 (Fig. 3e). Increasing the probability of amplification increases conflict, Fig. 3f shows that at $p$ = 0.5 and 0.8, conflict reaches more than 1.0.

## Countering extreme polarization

SOAM suggests that opinion amplification, even with low occurrence, and especially with low amplification can cause extreme polarization in the population. Given that such polarization is also evident in real world social networks, in this section we examine potential methods to counter, prevent, or even reverse extreme polarization. Recent research on polarisation mitigation and opinion control suggests various approaches, for example, Musco et al. studied ways to minimise polarisation and disagreement in social networks^27^, Garimella et al. suggests controversy can be reduced by connecting opposing views^28^, Rossi et al. studied closed loops between opinion formation and personalised recommendations^29^, while Matakos et al. proposed a recommender-based approach to break filter bubbles in social media^30^. Here we examine two techniques in use today by social networks to see their effectiveness in our model.

### Countering method 1: the five-strike system

A common strategy used by online social networks is to stop users from posting after a number of offenses that disobey their rules. (For example, Twitter’s medical misinformation policy (https://help.twitter.com/en/rules-and-policies/medical-misinformation-policy) has a strike system, each violation of the policy count as a strike, and 5 or more strikes results in a permanent suspension of the Twitter account.) We implement this policy by detecting amplified posts and if a user amplifies more than 5 times, they are no longer allowed to post further. To study the effect, we add a max amplify parameter, so that each agent can only amplify 5 times. Once they exceed this number, they are removed. In order to keep the population constant, we replace a removed individual with a new individual with the same default probabilities and random opinion.

We ran the model with the same amplification settings in the previous section, timesteps $t$ = 400, number of nodes $n$ = 100, and average links per node $k$ = 5, with the proportion of the population who are amplifiers $\pi$ = 0.5, amplification probability $p$ = 0.5, amplification strength $s$ = 0.5, for confidence thresholds $\varepsilon$ = 0.2 and 0.8. We compare a normal run of SOAM with a run that uses the maximum amplify intervention, both starting from the same random seed to ensure the composition of the initial populations are identical.

**Figure 4:**
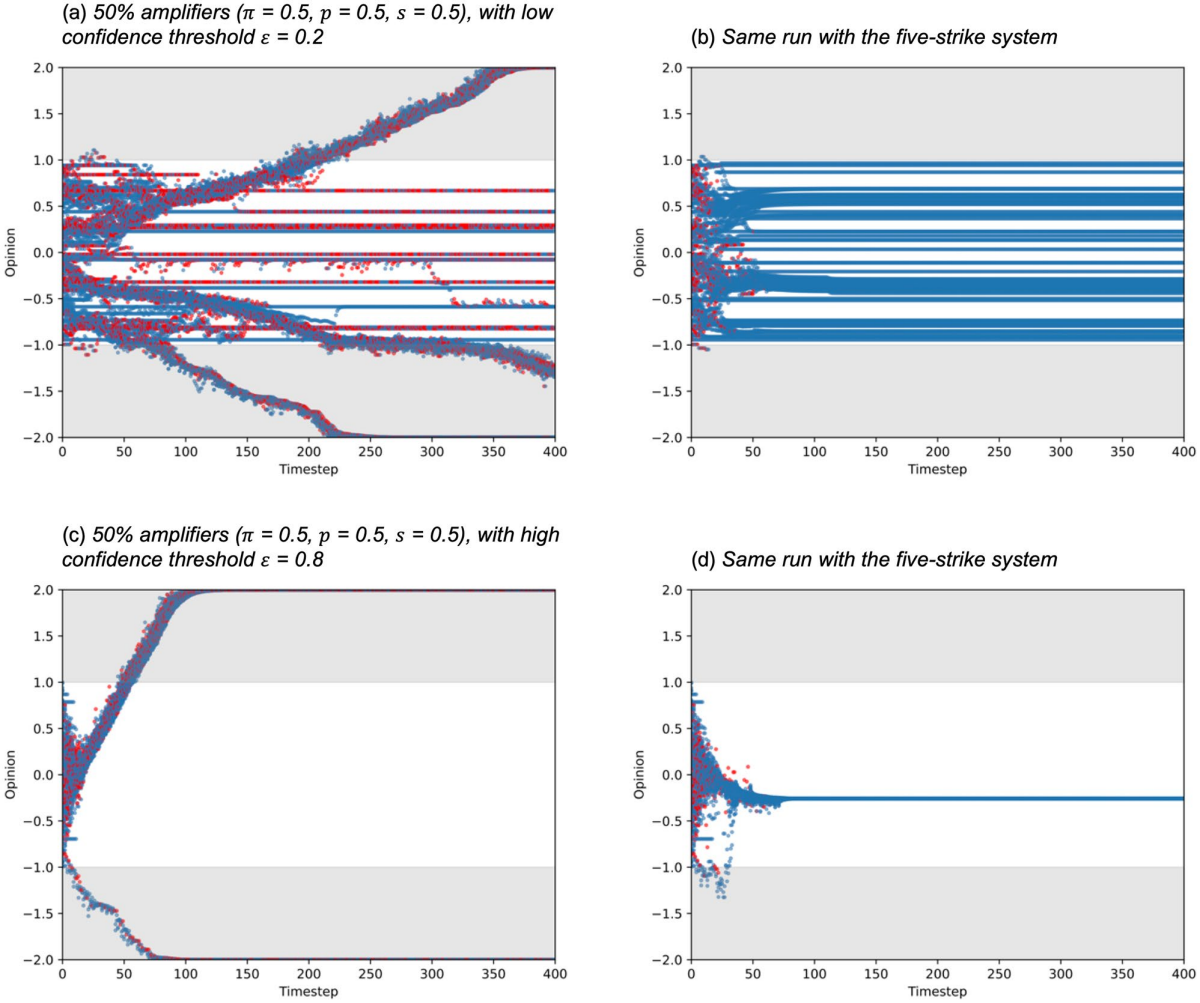
Opinions of individuals starting from a random distribution [−1.0, 1.0] with corresponding conflict plots. Red dots denote individuals who are amplifying in that timestep. Grey areas indicate opinions outside the initial opinion range.

While extreme polarization occurred in Fig. 4a, after applying the curbing method, Fig. 4b shows that extreme polarization no longer occurs. The same results can be seen for Fig. 4c versus Fig. 4d.

### Countering method 2: disseminating balanced opinions

A second approach to countering extreme opinions on social networks is for institutions to disseminate balanced opinions about any given topic. We model this by introducing an additional 5 random external opinions at every other timestep, which are randomly spread across the initial range [−1.0, 1.0], representing a normal range of non-polarized opinions. Every individual has access to this same set of opinions, every other timestep, and the same confidence threshold aspect applies, where people are only influenced by opinions that are within the confidence threshold. This simulates institutions exercising correctional behaviors of countering misinformation by publicly disseminating less polarized opinions across the original range of “normal” opinions. For example, the Science Media Centre (https://www.sciencemediacentre.org/about-us/) explicitly aims to disseminate “accurate and evidence-based information about science and engineering through the media, particularly on controversial and headline news stories when most confusion and misinformation occurs”. We ran the model with the same settings as before, timesteps $t$ = 400, number of nodes $n$ = 100, and average links per node $k$ = 5, with the proportion of the population who are amplifiers $\pi$ = 0.5, amplification probability $p$ = 0.5, amplification strength $s$ = 0.5, for confidence thresholds $\varepsilon$ = 0.2 and 0.8. Again, we compare a normal run of SOAM with a run that uses the intervention, both starting from the same random seed to ensure the composition of the initial populations are identical.

**Figure 5:**
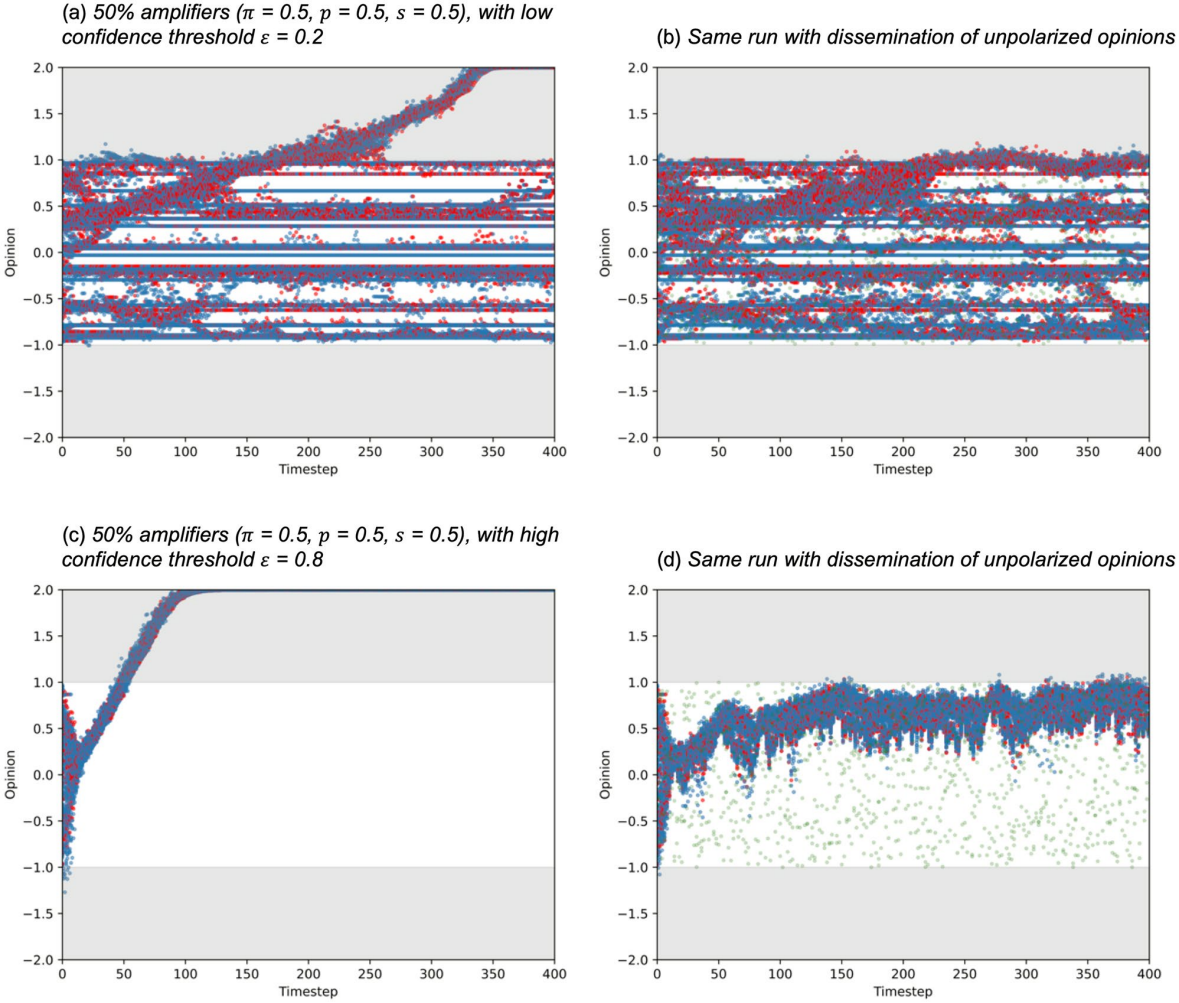
Opinions of individuals starting from a random distribution [−1.0, 1.0] with corresponding conflict plots. Red dots denote individuals who are amplifying in that timestep. Green dots denote external unpolarised opinions.

While extreme polarization occurred in Fig. 5a,b shows that extreme polarization no longer happens, although some opinions are slightly outside the original. The same results can be seen for Fig. 5c versus Fig. 5d. This shows that with a small but consistent intervention, extreme polarization can be curbed.

## Discussion

Research has previously shown that extreme polarization can be caused by strong external events impacting populations^2^. Our model suggests another factor: that extreme polarization can be caused by individuals simply amplifying their own opinions. We demonstrate for the first time that this simple idea results in extreme polarization. SOAM shows us that some common trends in recent communication amongst a minority can affect entire populations. Whether spin, sensationalism, clickbaits, hype, sentiment polarity, or even “fake news”, when opinions are amplified by a few, extreme polarization of many can result. This finding is consistent despite network models (the connections between individuals). In our experiments, we used a random network (specifically the Erdös-Rényi model), as it is most commonly used in existing literature to model social networks^26^. When we use SOAM with other network models that represent plausible network structures for online social networks: scale-free network^31^ and Barabasi-Albert network^32^, we find consistent results: extreme polarization occurred with all of them. Likewise, when we increase the number of connections *k* (i.e., to model online networks where people have numerous connections) we observe the same result.

Extreme polarization can cause several harmful effects. We explored two methods to address polarization caused by opinion amplification: preventing individuals from amplifying more than five times, and ensuring a consistent communication of opinions with sentiments that fall in a normal range. Both approaches showed that polarization can be curbed. This result is consistent with real world findings. For example, one study showed that Fox News viewers who watched CNN instead for 30 days became more skeptical of biased coverage^33^.

We reran the five-strike curbing method on the other types of networks and found that it curbed extreme polarization equally effectively. When we reran the disseminating unpolarized opinion method, we found that the Barabasi-Albert network^32^ requires either more frequent dissemination (e.g., daily) or higher number of messages (e.g., instead of 5 messages, 10, or 20), and sometimes both depending on the runs, to be effective—more connected individuals receive more extreme opinions and therefore may need stronger normal-range messaging to counter this.

It is always tempting for us to speak louder to be heard in a crowd. But when some begin to shout, others feel they must also shout. And when everyone attempts to out-shout everyone else, the result can be a screaming mob, all vocalizing at the top of their lungs. In a social network, the volume of sentiment can become amplified in the same way, which can result in groups with extreme polarized opinions. But this form of polarization is participatory and voluntary. If we choose to temper our expressed opinions, if we lower our voices and speak normally instead of screaming, then perhaps we might help provide that much-needed balance of normal sentiment to society, helping curb extremism for all.

## Data Availability

Data and code are available at http://www.cs.ucl.ac.uk/staff/S.Lim/polarization.
