# Publisher Correction: Opinion amplification causes extreme polarization in social networks

**DOI:** 10.1038/s41598-022-25620-5

**Published:** 2022-12-20

**Authors:** Soo Ling Lim, Peter J. Bentley

**Affiliations:** 1 Department of Computer Science, University College London, London, UK; 2 Autodesk Research, London, UK

Correction to: *Scientific Reports* 10.1038/s41598-022-22856-z, published online 28 October 2022.

The original HTML version of this Article contained an error in Figure 2, where panels (e) and (f) did not display correctly.

**Figure 2:**
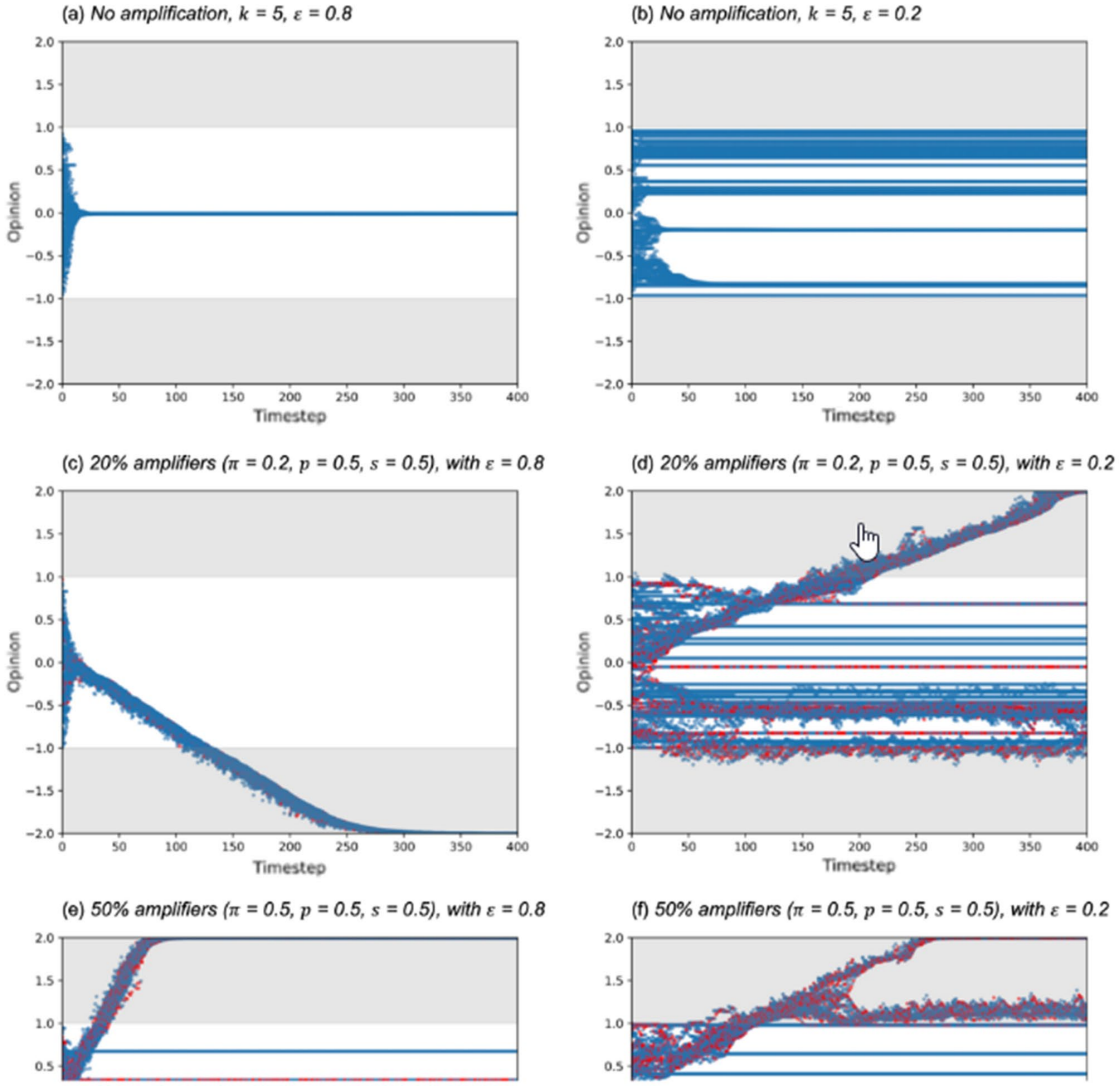
Opinions of individuals starting from a random distribution [− 1.0, 1.0] under a range of conditions. Red dots denote individuals who are amplifying in that timestep. Grey areas indicate opinions outside the initial opinion range. Y-axis shown from − 2.0 to 2.0 (double the initial opinion range) for clarity; in (**c**) to (**f**), opinions exceed this range and become even more extreme.

The original Figure 2 and accompanying legend appear below.

This error has now been corrected in the HTML version of the Article; the PDF version was correct from the time of publication.

